# Sickness and Death

**Published:** 1879-11

**Authors:** 


### SICKNESS AND DEATH.

Statistics seem to show that in an ordinary community there are twenty-eight persons sick where one dies, and that at the very least one half of this sickness is easily preventible. The number of persons dying out of one thousand of all ages and sexes in one year, is called the "Death-Rate;" this varies according to locality, customs, etc. In the most notorious districts in England it is thirty-six persons out of a thousand. In New-York city, in 1859, the reported death-rate was thirty-three in a thousand. The death-rate of all England is twenty-two; of the United States, twenty-four. But if a thousand men are taken—as the greatest mortality is among children—the death-rate is greatly reduced. The death-rate of the "Household Cavalry" is a little over eleven per thousand; of the "Foot Guards" twenty. Why this great difference of nearly one half? The cavalry are out of doors all the time; the guards are in-doors, sitting, sleeping, lounging, smoking, and breathe a vitiated atmosphere—showing that sitting still out of doors in the coldest winter weather, as is the official duty of the cavalry, for hours together, is not as destructive of health and life as sitting around in well-warmed barracks, breathing a contaminated atmosphere. The lowest death-rate, ascertained with any certainty, in the world, is in the isolated Islands of Faroe, where only fifteen persons out of a thousand die in a year. Therefore whatever number over fifteen die out of a thousand in any one year, is that much greater mortality than there ought to be. According to the admirable and able report of Daniel E. Delavan, Esq., City Inspector of New-York, there were 22,117 deaths during 1861, in a population of say 850,000, or 26 as the death-rate, being eleven per thousand more than it ought to be; hence there were 9350 unnecessary deaths in New-York city during 1861. Surely every family should read and practice the precepts of a journal like this, the chief object of which has been from the first, to show how to avoid sickness.

